# Poor Responses to Interferon-Beta Treatment in Patients with Neuromyelitis Optica and Multiple Sclerosis with Long Spinal Cord Lesions

**DOI:** 10.1371/journal.pone.0098192

**Published:** 2014-06-02

**Authors:** Kai-Chen Wang, Kuan-Hsiang Lin, Tzu-Chi Lee, Chao-Lin Lee, Shao-Yuan Chen, Shyi-Jou Chen, Li-Te Chin, Ching-Piao Tsai

**Affiliations:** 1 Department of Neurology, Cheng Hsin General Hospital, Taipei, Taiwan; 2 Neurological Institute, Taipei Veterans General Hospital, Taipei, Taiwan; 3 National Yang-Ming University, School of Medicine, Taipei, Taiwan; 4 Faculty of Public health, Kaohsiung Medical University, Kaohsiung, Taiwan; 5 Department of Neurology, I-Lan Hospital, I-Lan, Taiwan; 6 Section of Hyperbaric Oxygen Medicine, Cardinal Tien Hospital, Taipei, Taiwan; 7 Department of Microbiology and Immunology, National Defense Medical Center, Taipei, Taiwan; 8 Department of Microbiology & Immunology, National Chayi University, Chayi, Taiwan; Kyushu University, Japan

## Abstract

Interferon-beta (IFN-β) treatment may not be effective in neuromyelitis optica (NMO). Whether the poor response to IFN-β is related to long spinal cord lesions (LSCL) or the NMO disease entity itself is unclear. We evaluated the spinal cord involvement of patients with multiple sclerosis (MS) and NMO, as well as the response after receiving IFN-β. Forty-nine MS and 21 NMO patients treated with IFN-β for at least 2 years from 2002–2008 were enrolled in this study and the treatment response was analyzed 2 years post-treatment. In the study, spinal cord lesions were present in 57.1% (28/49) of the MS patients, of which 16.3% (8/49) presented spinal cord lesions longer than 3 vertebral segments (LSCL). Responses to IFN-β treatment were seen in 69.3% (34/49) of all the MS cases, of which the appropriate response rates were 76.1% (16/21) in MS patients without spinal cord lesions and 37.5% (3/8) in patients with LSCL. Only 14.2% (3/21) of NMO patients responded to IFN-β treatment. In conclusion, spinal cord lesion is common in MS patients in Taiwan. Both NMO and MS patients with LSCL had a poor response to IFN-β treatment. NMO patients had a worse response to IFN-β treatment than MS patients with LSCL, which shows that the crucial structural defect is something other than LSCL such as the elevated serum IL17 level in NMO compared to MS.

## Introduction

Long spinal cord lesions (LSCL) of 3 or more vertebral segments are quite a unique phenomenon in neuromyelitis optica (NMO). Unlike brain lesions which may have asymptomatic or silent lesions, lesions of the same size in the spinal cord definitely cause more severe symptoms/signs. NMO patients have a poor prognosis compared to those with multiple sclerosis (MS), since the spinal cord lesion itself is crucial in the disability scale. Nevertheless, spinal cord involvement is also quite common in MS in Asian countries [1], [2].

Interferon-β (IFN-β) treatment reduces the frequency of major MS attacks by 33% [3], and has been used to prevent exacerbation of relapsing-remitting MS (RRMS), including optic-spinal MS (OSMS) in Japan [4]. However, some reports have suspected that IFN-β treatment triggers severe exacerbation in patients with NMO or its spectrum form, especially when involved with LSCLs [5], [6]. Prior to 2008, NMO and MS were not so well distinguished; thus, IFN-β treatment was the first disease-modifying therapy for these patients.

There have been few studies reporting spinal cord lesions in large cohorts of MS patients and comparing IFN-β treatment responses in MS and NMO patients with spinal cord lesions. It is therefore important to verify the differences in the significance of spinal lesions in MS and NMO and their treatment responses to IFN-β. In order to determine whether poor response to IFN-β is related to the crucial LSCL or the NMO disease entity itself, we evaluated spinal cord involvement in Taiwanese patients with MS and NMO and correlated their MRI and anti-aquaporin-4 auto-antibodies (AQP4 Ab) with their response to IFN-β in the special clinic for MS at Taipei Veterans General Hospital (VGH) from 2002–2008.

## Materials and Methods

### Patient Enrollment

Eight-two patients with an initial disease-modifying IFN-β treatment from 2002–2008 at the special clinic for MS at Taipei VGH were collected for this observational study. Twelve dropped out within 2 years, leaving a total of 70 participants, including 49 MS and 21 NMO patients undergoing their first 2-year continued disease-modifying IFN-β treatment, consecutively enrolled in the study. The study was approved by the Institutional Review Board of Taipei VGH [(371)102-18], and the patients gave their written consent for their information to be stored in the hospital database and used for research purposes alone.

NMO was diagnosed based on Wingerchuk’s diagnostic criteria and MS was diagnosed using the 2005 revised McDonald’s criteria [7], [8]. In brief, optic neuritis and long spinal cord myelitis (>3 segments) constituted the diagnosis of NMO; the brain MRI of the patients was normal or did not meet the criteria for MS. Otherwise, the patient was diagnosed as having MS. Patients with MS or NMO treated with IFN-β less than 2 years, and those who had Sjögren’s syndrome or a systemic lupus erythematosus-related neurological disorder, recurrent myelitis, or recurrent optic diseases, were excluded.

The following were analyzed and correlated: gender, male/female ratio, baseline disease duration, clinical disease activity freedom, the MRI of the spinal cord and brain imaging at the beginning of treatment and the relapse imaging (analyzed blindly by Dr. CL Lee), Kurtzke’s Expanded Disability Status Scale score (EDSS) evaluations (the first EDSS evaluation at the beginning of treatment, relapse EDSS evaluation, and final evaluation 2 years post-treatment, all performed blindly by Dr. KH Lin), the annual relapse rate (ARR) during the 2-year treatment period, the subgroup (MS or NMO), existence of longitudinally extensive spinal cord lesions (LSCLs) longer than 3 vertebral segments or less than 3 vertebral segments, which were considered as short spinal cord lesions (SSCL), the disease duration, AQP4 Ab, and the response to treatment 2 years later. In detail, LSCL identification relied on lateral imaging that not only extended more than 3 vertebral segments but that also disclosed the whole cord involvement on the transverse MRI view, preventing the interpretation of the fusion of several small lesions into LSCLs, which are preferentially located on transverse spine MRI imaging that is commonly seen in MS.

Patients with a poor response to treatment were defined as those who had the following conditions: (1) 1 moderate/severe or 2 mild relapses or (2) no relapses but with more than 1 point compared to the baseline EDSS in each year of the 2 year-follow-ups. If the patients met a relapse-based non-response criterion in year 1 and a progression criterion in year 2, that was still considered as a poor response to treatment. Those who did not meet the above conditions in the follow-up were considered to have had a good response to treatment. A mild relapse was defined as not requiring corticosteroids, not having motor/cerebellar involvement, and having no effect on function and complete recovery; a moderate relapse was defined as requiring steroids, involving motor/cerebellar systems, affecting activities of daily living (ADL) and having an incomplete recovery at 3 months; a severe relapse was defined as requiring steroids and hospitalization, involving motor/cerebellar systems, and having severely affected ADL with incomplete recovery at 6 months [9], [10]. The poor responses to treatment endpoint included EDSS changes and relapse numbers measures because these patients with demyelinating disease might experience secondary progressive neurological deterioration even without a clinical relapse. Low-dose oral glucocorticoid treatment was added to IFN-β in patients who were considered as having a poor response during follow-up. If there was still a poor response with combined glucocorticoid and IFN-β use, the physician would add Azathioprine or stop IFN-β treatment in refractory NMO or in MS patients with LSCL (*Table S1*).

### Magnetic Resonance Imaging Study Method

All patients underwent MRI on a 1.5-T Scanner (Avanto, Siemens, Erlangen, Germany), using an 8-channel phased-array head Matrix coil attached to the neck Matrix coil in order to have a higher signal-to-noise ratio at the cervical region. The conventional MRI protocol included: sagittal STIR images (repetition time [TR]: 4170 ms; echo time [TE]: 87 ms; field of view [FOV]: 250 mm; matrix: 256×320; with a 10% gap), axial T2* (TR: 606 ms; TE: 18 ms; FOV: 200 mm; matrix: 192×320; 30 slices with 3 mm thickness and a 30% gap) and sagittal T1 (TR: 500 ms; TE: 9 ms; FOV: 220 mm; matrix: 320×240; 12 slices with 3 mm thickness and a 30% gap) after administration of contrast medium (dimeglumine gadolinium, 0.1 mmol/kg; Schering AG, Berlin, Germany).

### Anti-AQP4 Autoantibody Study Method

Blood samples were drawn after any previous episode of more than 3 months, i.e., during disease remission. Sera were harvested and stored at −20°C. Serum AQP4 Ab was detected using the cell-based assay with AQP4-transfected HEK293 cells described by Matuskoa et al [11].

### Enzyme-linked Immunoassay (ELISA) Determination for Serum Cytokines

Blood samples were drawn at the beginning of the study. Sera were harvested and stored at −20°C. Detection of the content of the cytokines (IL-17, and INF-γ) was performed using an ELISA kit (R & D Systems Inc., Minneapolis, MN, USA) according to the instructions of the manufacturer. The concentrations of cytokines (IL-17, and INF-γ) were calculated based on the standard curve.

### Statistical Analyses

We categorized the patients as having an ‘appropriate response’ or ‘poor response’ based on the above description. First, we compared disease progression between the appropriate and poor response patients using the t test. The main interest outcomes were change in EDSS and annual relapse rate from the EDSS 2 years post-IFN-β treatment (EDSSp) to baseline. Second, focusing on the outcome of EDSSp, all cases were analyzed using analysis of covariance (ANCOVA) to reveal the main and interactive effects of the groups: MS without spinal cord involvement (MS-NSCL), MS with LSCLs (MS-LSCL), MS with SSCL (less than 3 segments) (MS-SSCL), NMO-LSCL without AQP4 Ab (NMO-LSCL AQP4^−^) and NMO LSCL with AQP4 Ab (NMO-LSCL AQP4^+^), and the response to IFN-β treatment (appropriate or poor) by controlling the baseline EDSS (EDSSb), sex and age. Scheffe post-hoc tests were performed to find the significant mean difference of pair-wise groups. There were 3 groups in the cytokine assay: MS-LSCL, NMO and healthy groups. Statistical analysis was performed with a nonparametric Kruskal-Wallis test with Bonferroni-Dunn’s correction to assess differences between groups. The ANCOVA test was used to analyze how much these variables determined the EDSSp. Analyses were carried out using the SAS 9.1.

## Results

### Spinal Cord Lesions, AQP4Ab Status and Treatment Response in MS and NMO Patients

None of the MS patients were AQP4 Ab positive. Spinal cord lesions were present in 57.1% (28/49) of conventional MS cases, of which 16.3% (8/49) presented LSCL lesions and 40.8% (20/49) had SSCL lesions. Treatment response was seen in 69.3% (34/49) of all MS cases, including 76.1% (16/21) of MS-NSCL patients, 37.5% (3/8) of LSCL patients and 75% (15/20) of SSCL patients. The 8 NMO patients with positive AQP4 Ab showed no response to treatment, but 23% (3/13) of those with negative AQP4 Ab did show a response. The total response rate for NMO patients was 14.2% (3/21) (Table 1).

**Table 1:** Demographic statistics of all participants.

Group	Male/Female	Response	N	ResponderRate (%)	Age	BaselineEDSS	Post-RxEDSS	BaselineARR	ARRduringRX	DiseaseDurationMonths	Clinical diseaseactivityfreedom (N)
MS-NSCL	3/18	No	5	76.0	47.00±12.19	1.60±0.55	3.10±0.55	1.20±0.45	1.58±0.79	20.60±17.08	0
		Yes	16		35.19±9.47	0.91±0.66	0.97±1.12	1.19±0.44	0.98±0.80	31.19±19.15	11
MS-LSCL	1/7	No	5	37.5	39.80±12.36	3.80±0.84	6.20±1.57	1.20±0.45	1.45±0.18	18.40±6.07	0
		Yes	3		39.67±10.60	1.83±1.26	2.17±1.44	1.17±0.29	0.70±0.26	26.67±12.86	1
MS-SSCL	2/18	No	5	75.0	32.00±12.35	2.80±0.45	4.30±1.10	1.20±0.45	1.42±1.23	22.40±8.41	0
		Yes	15		32.80±12.68	1.27±0.56	1.37±0.64	1.10±0.39	1.02±0.75	23.80±14.13	9
NMO LSCL AQP4^−^	0/13	No	10	23.0	39.70±9.04	4.40±0.70	7.10±1.35	1.20±0.35	1.46±0.98	21.80±10.00	0
		Yes	3		32.00±8.72	1.67±0.58	2.00±1.80	1.33±0.58	1.08±0.80	24.67±11.02	1
NMO LSCL AQP4^+^	0/8	No	8	0.0	41.00±10.43	3.50±1.20	4.88±1.33	1.03±0.07	1.09±0.64	26.50±10.94	0
		Yes	0		–	–	–	–	–	–	

Abbreviation: MS without spinal cord involvement (MS-NSCL), MS with long spinal cord lesions (MS-LSCL), MS with short spinal cord lesions (less than 3 segments) (MS-SSCL), NMO-LSCL without AQP4 Ab (NMO-LSCL AQP4^−^) and NMO LSCL with AQP4 Ab (NMO-LSCL AQP4^+^), Rx: interferon-β therapy, ARR: annual relapse rate.

### Comparison of Disease Progression between Appropriate and Poor Response Patients

The EDSS 2 years post-IFN-β treatment (EDSSp) (3.10±0.55, mean ± SD) for the MS NSCL patients with a poor response was significantly higher than that at baseline (1.60±0.55, P = 0.009). However, the EDSSp (0.91±0.66) of the MS NSCL patients with a good response was similar to that at baseline (0.97±1.12, P = 0.718). The change in EDSS (1.50±0.71) of the MS NSCL patients with a poor response was significantly greater than that of the patients with a good response (0.06±0.68, P<0.001). Similar disease progression results represented by the increase in EDSS were also found in the MS LSCL, MS SSCL and NMO LSCL AQP4^−^ groups. In addition, the disease progression results represented by the change in ARR between the patients with an appropriate and a poor response were all consistent (Table 2).

**Table 2:** Comparison of disease progression represented by increase in EDSS and annual relapse rate.

Group	Score	Appropriate Response			Poor Response			P value^2^
		Baseline	Post-Rx	P value^1^	Baseline	Post-Rx	P value^1^	
MS NSCL		N = 16			N = 5			
	EDSS	0.91±0.66	0.97±1.12	0.718	1.60±0.55	3.10±0.55	0.009	
	Change in EDSS	0.06±0.68			1.50±0.71			<0.001
	ARR	1.19±0.44	0.98±0.80	0.098	1.20±0.45	1.58±0.79	0.342	
	Change in ARR	−0.21±0.48			0.38±0.79			0.053
MS LSCL		N = 3			N = 5			
	EDSS	1.83±1.26	2.17±1.44	0.423	3.80±0.84	6.20±1.57	0.002	
	Change in EDSS	0.33±0.58			2.40±0.74			0.006
	ARR	1.17±0.29	0.70±0.26	0.250	1.20±0.45	1.45±0.18	0.336	
	Change in ARR	−0.47±0.50			0.25±0.51			0.101
MS SSCL		N = 15			N = 5			
	EDSS	1.27±0.56	1.37±0.64	0.531	2.80±0.45	4.30±1.10	0.009	
	Change in EDSS	0.10±0.0.60			1.50±0.71			<0.001
	ARR	1.10±0.39	1.02±0.75	0.629	1.20±0.45	1.42±1.23	0.579	
	Change in ARR	−0.08±0.61			0.22±0.82			0.393
NMO LSCL AQP4^−^		N = 3			N = 10			
	EDSS	1.67±0.58	2.00±1.80	0.830	4.40±0.70	7.10±1.35	<0.001	
	Change in EDSS	0.33±2.36			2.70±1.32			0.042
	ARR	1.33±0.58	1.08±0.80	0.225	1.20±0.35	1.46±0.98	0.312	
	Change in ARR	−0.25±0.25			0.26±0.76			0.292
NMO LSCL AQP4^+^		N = 0			N = 8			
	EDSS	–	–	–	3.50±1.20	4.88±1.33	0.003	
	Change in EDSS	–	–	–	±			–
	ARR	–	–	–	1.03±0.07	1.09±0.64	0.789	
	Change in ARR	–	–	–	±			–

1 Baseline vs. post-Rx.

2 Appropriate response vs. Poor response.

ARR: annual relapse rate.

Rx: IFN-β treatment.

### Different Patient Groups and Initial EDSS Determined the 2-year EDSS after IFN-β Treatment

The ANCOVA was used to test the main effect of treatment response and study group, including their interaction (Group × Response), by controlling sex, age and EDSSb (Table 3). An obvious main effect of response on EDSSp was found: on average, the non-responding patients had a higher EDSSb than the responding patients (Table 2). When controlling EDSSb, no significance was found for sex and age, but the different groups (MS-NSCL, MS-LSCL, MS-SSCL, NMO-LSCL AQP4^−^ and NMO-LSCL AQP4^+^) determined EDSSp.

**Table 3:** Analysis of covariance in EDSSp post-interferon-β therapy.

Source	Degree of freedom	F Value	P
Baseline EDSS	1	31.68	<0.0001
Sex	1	0.41	0.5239
Age	1	2.11	0.152
Group	4	3.06	0.0234
Response	1	24.6	<0.0001
Group × Response	3	0.96	0.4188

### Comparisons between Groups On the Mean of EDSSp Post-IFN-β Treatment

The mean EDSSp of NMO-LSCL AQP4^−^ patients were higher than for MS-SSCL or MS-NSCL patients. The mean EDSSp in each group, including the NMO-LSCL AQP4^−^, NMO-LSCL AQP4^+^ and MS-LSCL, showed no significant difference. The mean EDSSp of NMO-LSCL AQP4^+^ patient was higher than that of MS SSCL patients. The mean EDSSp of NMO-LSCL AQP4^+^ patients and those with MS-LSCL were not significant. The EDSSp of MS-LSCL patient was higher than that for patient with MS-SSCL or MS-NSCL. The mean EDSSp of MS-SSCL and MS-NSCL patients showed no significant difference (Table 4).

**Table 4:** Comparisons of the mean of EDSSp post-interferon-β therapy between groups.

Comparisons		N(Group 1/Group 2)	Mean(Group 1/Group 2)	Difference between means	95% CI ofmean difference
Group 1	Group 2				
NMO LSCL AQP4^−^	NMO LSCL AQP4^+^	13/8	5.92/4.88	1.05	−0.26∼2.36
NMO LSCL AQP4^−^	MS LSCL	13/8	5.92/4.69	1.24	−0.07∼2.54
NMO LSCL AQP4^−^	MS SSCL	13/20	5.92/2.10	3.82	2.79∼4.86
NMO LSCL AQP4^−^	MS NSCL	13/21	5.92/1.48	4.45	3.42∼5.47
NMO LSCL AQP4^+^	MS LSCL	8/13	4.88/4.69	0.19	−1.27∼1.64
NMO LSCL AQP4^+^	MS SSCL	8/20	4.88/2.10	2.78	1.56∼3.99
NMO LSCL AQP4^+^	MS NSCL	8/21	4.88/1.48	3.40	2.19∼4.61
MS LSCL	MS SSCL	13/20	4.69/2.10	2.59	1.37∼3.80
MS LSCL	MS NSCL	13/21	4.69/1.48	3.21	2.00∼4.42
MS SSCL	MS NSCL	20/21	2.10/1.48	0.62	−0.29∼1.53

### Grouping and EDSSp 6.5+

According to Fisher’s exact test, the percentage of EDSSp 6.5+ by group was significantly different (P<0.001). The percentage of EDSSp 6.5+ of the MS-LSCL, NMO-LSCL AQP4^−^ and NMO-LCL AQP4^+^ groups was not significantly different. The percentage of EDSSp 6.5+ of the MS LSCL, NMO LSCL AQP4^−^ and NMO LSCL AQP4^+^ groups was significantly higher than that for the MS-NSCL/MS-SSCL group. (Table 5).

**Table 5:** Percentage of EDSSp 6.5+ by group.

Group	Number of EDSSp less than 6.5	Number of EDSS 6.5+	Percentage of EDSSp 6.5+
MS LSCL	5	3	37.5
MS NSCL/MS SSCL	21/20	0	0.0
NMO LSCL AQP4^−^	6	7	53.85
NMO LSCL AQP4^+^	6	2	25.00

## Discussion

In our study, spinal lesions were found in 28/49 (57.1%) patients in the MS group, among whom, 8/49 (16.3%) had LSCL and 20/49 (40.8%) had SSCLs. Spinal cord lesions are common in our MS patients, similar to findings in Western studies [12]–[16]. However, all of our NMO patients showed LSCL involvement. Furthermore, an increased EDSSp was found in 3 groups, including NMO-LSCL AQP4^−^, NMO-LSCL AQP4^+^ and MS-LSCL. LSCL determined severe disability whether in MS or NMO, and resulted in a poor response to IFN-β treatment. All of the poor responders were prescribed oral prednisolone at the end of follow-up. (*Table S1*).

In Western MS series reports, spinal cord lesions usually spanned less than 2 vertebral segments and occupied less than one-half of a spinal cross-section, preferentially involving the peripheral white matter. LSCL extending beyond 3 vertebral segments was rarely seen in classical MS patients in Western populations (only 3% in 1 report) [14]. In addition, these MS-LSCL patients were negative for serum NMO-IgG antibody testing [17]. Our MS-LSCL patients all showed negative AQP4 Ab findings, as well.

Studies from Asia report LSCL is more frequently observed in about a quarter of MS cases and half of OSMS cases, reflecting the severe spinal cord damage seen in East Asian MS patients [18], [19]. Our MS series showed an even higher percentage of LSCL, not only involving more than 3 vertebral segments, but also centrally located, as seen in the spine MRI. This could explain why MS-LSCL patients have more disability, and show a poor response to treatment compared to MS-SCL patients.

In our study, IFN-β treatment for MS had an excellent effect, with about 69.3% of all MS patients responding. Treatment reduced the frequency of major MS attacks by 50%, as reported from Japan, and has been used to prevent exacerbation of RRMS, including OSMS [4], [20]. Based on these observations, it was concluded that treatment has comparable effects on patients with OSMS and MS in Asia. However, our MS-LSCL patients had a poor response to treatment, with only 37.5% responding, compared to 76% of MS patients without spinal cord lesions.

We also checked the serum cytokine expression in these MS-LSCL and NMO patients and found a similarly high expression of IL-17 in both groups (*Table S2*). RRMS patients with high serum IL-17 levels do not respond well to IFN-β therapy [21]. Our MS-LSCL and NMO patients had a poor response to treatment, which might be related to the elevated IL17, indicating a much more powerful immuno-modulating agent for down-regulating the Th17 response is needed for these patients [22].

Our NMO patients showed only a 14.2% response rate, which is lower than that of the MS patients and indicates a less effective response. There were no significant differences in our NMO cases with or without AQP4 Ab. Two Japanese reports indicated that IFN-β treatment was not effective in reducing the relapse number and the disability progression, and concluded that IFN-β treatment does not appear to be effective for preventing relapse in NMO, likely due to differences in the immune-pathogenesis between NMO and MS [23], [24]. Some studies suggest that treatment may trigger severe exacerbation in patients with the NMO spectrum [25], and also illustrate an increase in AQP4 Ab associated with such treatment [5].

However, our study included only a small number of MS-LSCL patients (8 patients), and they were much more disabled than other MS patients at baseline. It is therefore difficult to determine if MS with LSCL is just severe MS and therefore relatively IFN-beta unresponsive or if LSCL is a marker of poor IFN-beta response in MS, independent of other baseline covariates (e.g., EDSS, disease duration, prior treatments). Despite the above, we still found that MS-LSCL patients showed a poor response to IFN-β treatment.

Since NMO attacks are sometimes very severe, regardless of therapy, and unlike brain lesions which may have asymptomatic or silent lesions, spinal lesions usually cause severe disability with just a single episode of attack, our findings show a worse response rate in NMO than in MS-LSCL. All of the cases with a relapse EDSS greater than 6.5 were NMO, and despite having the same LSCL lesions, MS-LSCL showed milder symptoms and signs than NMO-LSCL. This demonstrates that the poor response of NMO patients to IFN-β is not only due to the LSCL, but that a special NMO entity and pathophysiology may also play an important role in the poor response to IFN-β treatment in NMO.

## Conclusion

Long spinal lesions are not rare among our MS patients. The poor response to IFN-β is related to the LSCL anatomic lesions in both MS and NMO, which cause severe symptoms and signs. However, NMO patients had a much worse response to IFN-β, indicating that factors other than the spinal lesion are crucial in the disability of NMO patients. The NMO disease entity itself is another critical consideration related to the poor response to IFN-β treatment of NMO patients.

## Supporting Information

Table S1 Demographic statistics of the participants with a poor response to treatment.
(DOCX)

Table S2 Cytokine levels in different groups.
(DOCX)
