# Supplementary material for: Poor Responses to Interferon-Beta Treatment in Patients with Neuromyelitis Optica and Multiple Sclerosis with Long Spinal Cord Lesions
Source: PLoS One. 2014 Jun 2;9(6):e98192. doi: 10.1371/journal.pone.0098192 (PMC4041653; doi:10.1371/journal.pone.0098192)
Supplement: Table S1 — Demographic statistics of the participants with a poor response to treatment. (DOCX) [file pone.0098192.s001.docx]

Table S1 Demographic statistics of the participants with a poor response to treatment

| Group | Male/Female | Steroid Responder Rate (%)^1^ | Steroid Combined Rx months | Azathioprine Responder rate (%)^2^ | Azathioprine Combined Rx months | Stop IFN-β Responder Rate (%) | Stop IFN-β months |
| --- | --- | --- | --- | --- | --- | --- | --- |
| MS-NSCL | 1/4 | 100.0 (5/5) | 12 |  |  |  |  |
| MS-LSCL | 0/5 | 60.0 (3/5) | 12.50±2.50 |  |  | 100 (2/2) | 11.00±1.41 |
| MS-SSCL | 0/5 | 100.0 (5/5) |  |  |  |  |  |
| NMO LSCL AQP4^-^ | 0/10 | 30.0 (3/10) | 14.50±3.50 | 40 (2/5) | 5.00±1.53 | 100 (3/3) | 16.00±2.00 |
| NMO LSCL AQP4^+^ | 0/8 | 37.5 (3/8) | 14.50±5.00 | 20.0 (1/5) | 7.70±2.72 | 100 (4/4) | 16.67±1.53 |

^1^ Prednisolone 10 mg

^2^ Azathioprine 100 mg
