# Supplementary material for: Poor Responses to Interferon-Beta Treatment in Patients with Neuromyelitis Optica and Multiple Sclerosis with Long Spinal Cord Lesions
Source: PLoS One. 2014 Jun 2;9(6):e98192. doi: 10.1371/journal.pone.0098192 (PMC4041653; doi:10.1371/journal.pone.0098192)
Supplement: Table S2 — Cytokine levels in different groups. (DOCX) [file pone.0098192.s002.docx]

Table S2 Cytokine levels in different groups

| Cytokine levels | NMO | MS-LSCL | Healthy | **P**-**value^a^** | **Post-hoc test^b^** |
| --- | --- | --- | --- | --- | --- |
| IFN r | 7.52 ± 1.30 | 3.1 ± 0.80 | 0.21± 0.98 | <0.001* | N>M>H |
| IL-17 | 30.15 ± 11.70 | 32.61 ± 17.12 | 2.71 ± 1.48 | 0.023* | N=M>H |

^a^ Kruskal-Wallis test was used for analysis. *, significantly different among the 3 groups (*P* < 0.05).

^b^ Mann-Whitney U test was used for the post-hoc test. N, Neuromyelitis optica patients; M, multiple sclerosis patients with long spinal cord involvement; H, healthy individuals. (P<0.0167)
